# Impact of the COVID-19 Pandemic on Clinical Pathways for Non-SARS-CoV-2 Related Diseases in the Lazio Region, Italy

**DOI:** 10.3390/ijerph19020635

**Published:** 2022-01-06

**Authors:** Maria Piane, Lavinia Bianco, Rita Mancini, Paolo Fornelli, Angela Gabriele, Francesco Medici, Claudia Battista, Stefania Greco, Giuseppe Croce, Laura Franceschetti, Christian Napoli, Mario Ronchetti, Paolo Anibaldi, Giorgio Banchieri

**Affiliations:** 1 Department of Clinical and Molecular Medicine, “Sapienza” University of Rome, Viale Regina Elena 291, 00161 Rome, Italy; rita.mancini@uniroma1.it; 2 Sant’Andrea University Hospital, Via di Grottarossa 1035-1039, 00189 Rome, Italy; christian.napoli@uniroma1.it (C.N.); panibaldi@ospedalesantandrea.it (P.A.); 3 Department of Public Health and Infectious Diseases, “Sapienza” University of Rome, Piazzale Aldo Moro 5, 00185 Rome, Italy; lavinia.bianco@uniroma1.it; 4 ASIQUAS (Associazione Italiana per la Qualità della Assistenza Sanitaria e Sociale), Largo Konrad Adenauer 1/B, 00144 Rome, Italy; fornellip26@gmail.com (P.F.); ronchetti.mario@gmail.com (M.R.); giorgio.banchieri@gmail.com (G.B.); 5 Department of Social Sciences and Economics, “Sapienza” University of Rome, Via Salaria 113, 00198 Rome, Italy; laura.franceschetti@uniroma1.it; 6 Azienda Sanitaria Locale (ASL) Frosinone District C and D, Via De Bosis-03043 Cassino, Via Piemonte, 03039 Sora, Italy; angela.gabriele1@virgilio.it; 7 San Camillo-Forlanini Hospital, Circonvallazione Gianicolense, 87, 00149 Rome, Italy; fam.medici@mac.com (F.M.); stefaniagreco@libero.it (S.G.); 8 Azienda Sanitaria Locale (ASL) RM 6 District of Mental Health and Pathological Addictions, Via Borgo Garibaldi 12, 00041 Albano Laziale, Italy; claudia.battista@gmail.com; 9 Internal Medicine Unit-“G. Mazzini” Hospital-ASL 4 Teramo-Piazza Italia 1, 64100 Teramo, Italy; gpp.crc@me.com; 10 Department of Medical Surgical Sciences and Translational Medicine, “Sapienza” University of Rome, Via di Grottarossa 1035/1039, 00189 Rome, Italy

**Keywords:** clinical pathway (CP), SARS-CoV-2, COVID-19, primary health care, health care system

## Abstract

Clinical pathways (CPs) are multidisciplinary clinical governance tools necessary for the care management of the patients, whose aim is to outline the best practicable path within a health organization related to an illness or to a complex clinical situation. The COVID-19 pandemic emergency has created the need for an organizational renewal of care pathways based on the principles of “primary health care” recommended by the WHO. In Italy, the Hospitals and Local Health Authorities (ASL) have tried to guarantee the continuity of non-deferrable treatments and the maximum safety of both patients and health professionals. This study analyzes the organizational and managerial responses adopted in pathology-specific care pathways to assess how CPs as diagnostic tools responded to the COVID-19 pandemic in the first two waves. Twenty-four referents of Operational Units (UU OO) from Hospitals (AO) and Local Health Authorities (ASL) of the Lazio Region (Central Italy) that apply four different CPs responded to a survey, which analyzes the managerial and organizational responses of CPs in regard to different contexts. Results show that the structural and organizational adjustments of the CPs have made it possible to maintain an adequate level of care for specific treatment processes, with some common critical aspects that require improvement actions. The adjustments found could be useful for dealing with new outbreaks and/or new epidemics in order to try to mitigate the potential negative impact, especially on the most vulnerable patient categories.

## 1. Introduction

Clinical pathways (CPs) are widely recognized as one of the main tools for designing and structuring care processes focused on patients’ needs, thus improving healthcare quality [1]. Moreover, well-organized CPs allow health care systems (HCS) to avoid the dispersion in the request of care made by the patients, as well as slowdowns in the response.

In Italy, the pandemic emergency started after the first case of COVID-19, diagnosed in Codogno (Lombardy) on 21 February 2020, causing a serious health crisis in a very short period of time [2]. Based on previous scientific evidence in the control of infectious diseases, several preventive measures were adopted and access to hospitals for non-urgent cases was severely reduced to preserve the care capacity of the HCS and protect both healthcare professionals and patients from infection [3,4,5,6]. Therefore, elective interventions and outpatient activities were postponed and all human and organizational resources of the HCS were dedicated almost exclusively to the management of patients with COVID-19 [7]. Consequently, there has been a reduction in hospitalizations [8], in the number of surgeries [9], and in diagnostic and therapeutic procedures [10,11]. Furthermore, during the first wave (February to June 2020), many health workers, especially doctors and nurses, acquired the disease, with consequent staff reductions due to long absences, which further impacted on the provision of services [12,13].

On 1 June 2020, the “Guidelines for the progressive reactivation of health services considered postponable during the COVID-19 emergency” [14] were published by the Ministry of Health, which opened a new phase, in which the need to ensure essential levels of care for patients suffering from non-COVID-19 diseases was highlighted. Different control measures, including priority access systems for vulnerable patients, were foreseen to allow a safer access to HCS, also thanks to specific methods of indoor environmental control measures that were applied beside the routine microbiological checks [15,16].

In addition, CPs were again available to patients and, in some cases, it was also necessary to remodel the CP to properly include remote health services.

**Figure 1:**
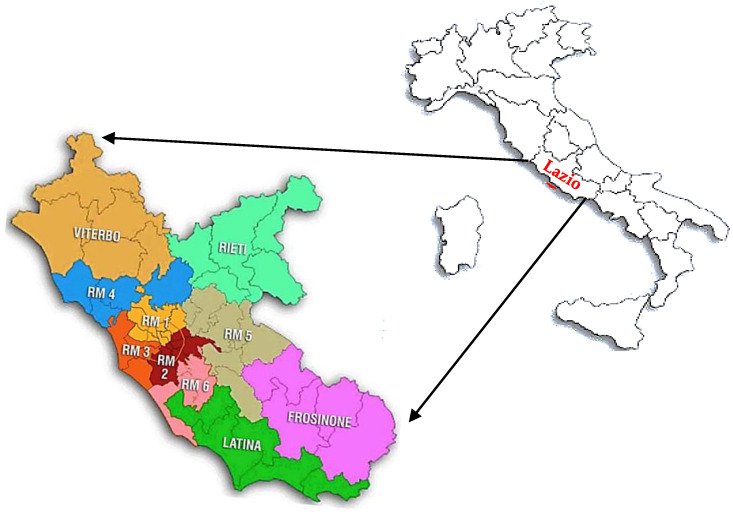
Geographic distribution of the centers that were invited to voluntarily participate in the survey by responding to the online questionnaire. RM1, University Hospital Sant’Andrea, Rome; RM3, Hospital San Camillo Forlanini, Rome; RM6, District of Mental Health and Pathological Addictions, Rome; ASL Frosinone District C and D.

The aim of the present study is to evaluate the impact of the pandemic on four CPs related to non-COVID-19 diseases (heart failure, hereditary breast-ovary cancers, autism spectrum disorders, and diabetes) in the Lazio Region (Central Italy) (Figure 1) during the first and second waves of the COVID-19 pandemic. Furthermore, it was evaluated if the CPs were able to implement the necessary organizational renewals and provide care in safe conditions.

## 2. Materials and Methods

A cross-sectional study was performed from 4 February to 24 February 2021 among the heads of 24 Operative Units (UU.OO.) that apply the four chosen CPs. The UU.OO. belonged to four different Local Health Authorities (ASL)/Hospitals (AO) and were divided in: five UU.OO. related to the heart failure CP; seven UU.OO. to the hereditary breast-ovarian cancers CP; six UU.OO. to the autism spectrum disorders (DSA) CP; and six UU.OO. to the Diabetes CP (Table 1 and Figure 1). Participants were invited to voluntarily take part to the survey by responding to an online questionnaire (Supplementary Material Table S1). The link to the web-based questionnaire was sent, via WhatsApp or by email, directly to the referents of the involved UU.OO.

The study was conducted anonymously following the provisions of the World Medical Association Declaration of Helsinki. The Ethical Committee of Sapienza University of Rome, Italy was acquired (RIF. CE 5773_2020).

### The Survey Questionnaire

The questionnaire, consisting of 37 items, was developed on the “Microsoft Forms” platform (Microsoft Office 365, 2021) by a multidisciplinary working group comprised of physicians and healthcare researchers not involved in the investigated CPs. The items of the questionnaire were designed based on guidance provided by government official documents, published literature, and best practices [1,5,17,18,19] and firstly validated by the opinions of a panel of experts composed of one oncologist, one geneticist, one epidemiologist, one psychologist and one expert in CPs.

The questionnaire was previously tested in a pilot study (data not published) in order to evaluate the questionnaire’s comprehensibility. The pilot sample of health care workers was asked to assign an intelligibility rating to each question on a 7-point scale (replying to the question: “Does the following sentence make sense to you?” in which 1: not meaningful and 7: very meaningful); a mean score > 5 per question was considered as the cut off for acceptability. For this purpose, the original questionnaire was modified: aside from the questions belonging to the standard questionnaire (SQQ), five additional questions (AQ) reporting grammatical and semantic errors were included in order to guarantee answer variability. SQQ reported a mean score for each question ≥ 5.85; AQ reported a mean score for each question ≤ 2. Therefore, the content of the questionnaire was considered clear to the readers.

The chosen platform allowed to invite users to respond through almost any web browser or mobile device, to see the results in real-time, to analyze the results and finally, to export the results in a file useful for further analysis. The survey could be completed within approximately 10 min.

The topics analyzed were divided into eight sections (Supplementary Material Table S1), allowing us to study the adaptability of the care pathways during both the first and second waves of the pandemic. The sections were:

1. Context analysis;
2. Patients access to care pathways/Operational Unit;
3. Impact on the treatment of non-COVID patients in the CPs;
4. Impact on the treatment of patients also SARS-CoV-2 infected in the CPs;
5. Impact of the COVID-19 pandemic on patient management;
6. Structural and organizational changes of the CP/UO;
7. Procedures and recommendations for healthcare professionals/users;
8. Training, information, and management of health workers in the pandemic era.

The eight sections investigated the adaptive capacities of the UU.OO. to the pandemic, in addition to the structural and the managerial changes, how the processes were modified, and any inadequacies of the organizational models reported. The questionnaire used “graded answers”, for each criterion, there were five possible answers: yes, enough, not enough, not at all, and not applicable, and each answer was connected to a percentage range (specifically: yes ≥ 75%; 51% ≤ enough ≤ 74%; 26% ≤ not enough ≤ 50%; and not at all ≤ 25%), and respondents were asked to indicate the verbal category that comes closest to their position.

Moreover, in order to have a comprehensive and objective report of how the Lazio Region dealt with the CP during the COVID-19 pandemic, a total score was calculated from the sum of the Likert scale values, where “yes” is equal to 4, “enough” to 3, “not enough” to 2, “not at all” to 1 and “not applicable” to 0 [20]. The means and standard deviations (SDs) of the bipolar 4-point Likert scales were calculated for each question of the survey. Means and SDs were also calculated for every section of the questionnaire (on its own and for each CP), and for each CP considering all the sections at the same time (Table 2 and Table 3).

This system allowed to put together and elaborate the findings into a numeric scale.

In order for these numbers to have significant meaning, we considered as independent variables the UU.OO. with each other and all the sections with each other, and we gave the same weight to all the questions.

It was considered as the cut off for an acceptable level of performance of the CP a mean score ≥ 1.80, and as the cut off for a good level of performance of the CP a mean score ≥ 2.99; a mean score < 1.80 was considered as a not acceptable level of performance.

Finally, for each item of the survey, we extrapolated the percentages of the five graded answers compared to the total number of answers given by the respondents (Table 3). Data analysis was performed with the use of Excel (Microsoft Office, 2019).

## 3. Results

The enrolled referents of the 24 UU.OO. applying the CPs replied to the online questionnaire aimed at verifying the impact of the COVID-19 pandemic on their activity. We analyzed five UU.OO. for the heart failure CP, seven UU.OO. for the hereditary breast-ovarian cancers CP, six UU.OO. for the autism spectrum disorders CP and six UU.OO. for the Diabetes CP.

All results are summarized in Table 2, Table 3 and Table 4, in Supplementary Table S2 and Supplementary Material SM1.

Using the scores obtained through the Likert scale, we calculated the mean score and its standard deviation for every CP with the following results: for the heart failure CP mean score 2.54 ± 0.17; for the hereditary breast-ovarian cancers CP mean score 3.19 ± 0.12; for the autism spectrum disorders CP mean score 2.81 ± 0.14, and for the Diabetes CP mean score 2.90 ± 0.25. Therefore, considering the established cut-offs, the heart failure CP, the autism spectrum disorders CP, and the Diabetes CP maintained an acceptable level of performance, while the hereditary breast-ovarian cancers CP maintained a good level of performance.

We also calculated the mean score and its standard deviation for each section of the survey, both in its entirety and for a single CP; the results are summarized in Table 2.

The results regarding each question are summarized in Table 3.

The most relevant findings, in each section, are:

1. Context analysis: only in 7 UU.OO. (29.2%) more than 75% of the patients accepted the treatment within the CP, the fear of being infected notwithstanding; compared to the same period of the previous year, during the first wave, there was a reduction in the treatment given within the CP in 91.7% of the UU.OO., as only 8.3% UU.OO. declared that the accesses remained stable (mean score 2.21 ± 1.04). By contrast, patient management stabilized in the second pandemic event according to 20 respondents (83.3%; 62.5% “yes” and 20.8% “enough”, with a mean score 3.38 ± 0.99).
2. Patient access to CP/UO: The CPs have been adapted to the pandemic setting by adopting security and social distancing measures in 91.7 and in 79.2% of the UU.OO. respectively (mean score 3.92 ± 0.28 and 3.71 ± 0.68, respectively). Patients who accepted treatment within the CP filled a preliminary pre-triage form in 83.3% of the UU.OO. (mean score 3.46 ± 1.22).
3. The impact on the treatment of NON-COVID patients in the clinical pathway: despite the COVID-19 pandemic, in the majority of the UU.OO. (66.7% “yes”, 20.8% “enough”) the access and the treatment were guaranteed in any case, and the canceled visits were rescheduled in 75.0% of the UU.OO. (58.3% “yes”, 16.7% “enough”). Overall, 58.3% (16.7% “yes”, 41.7% “enough”) of the UU.OO. used telemedicine in the form of remote monitoring to avoid the care interruption (mean score 2.25 ± 1.39) and 54.2% (20.8% “yes”, 33.3% “enough”) of the UU.OO. have adopted tele-assistance solutions for patient follow-up (mean score 2.25 ± 1.42).
4. The impact on the treatment of patients also infected with SARS-CoV-2 in the CPs: the CPs have not been shown to be adequate for the management of patients affected by SARS-CoV-2. In fact, no UU.OO. declared that they treated patients affected by COVID-19 within the care pathway/hospital ward answered “yes”, and only 2 (8.3%) answered “enough” (mean score 1.04 ± 0.84). It’s the only section that registered a not acceptable level of performance, both as a whole and for the single questions. Most of the enrolled healthcare settings were not integrated into the COVID Hospitals’ net, therefore 83.3% UU.OO. found the question if they had treated COVID-19 positive patients within a COVID ward not applicable to their setting (mean score 0.21 ± 0.50).
5. Impact of the COVID-19 pandemic on patient management: the SARS-CoV-2 screening test was routinely repeated during hospitalization in 45.8% of the UU OO, even if 45.8% found the question not applicable to their setting (mean score 1.96 ± 1.93), and in 91.7% of the UU.OO. the correct use of personal protective equipment (PPE) by staff and patients was monitored (mean score 3.71 ± 0.98).
6. Structural and organizational changes of the CP/UO: in 20 UU.OO. (83.3%; 58.3% “yes” and 25.0% “enough”) outpatient and/or surgical activities were guaranteed anyway (mean score 3.13 ± 1.36), and in 19 (79.2%; 37.5% “yes”, 41.7% “enough”) the timing in the transition of patients from one care setting to another was respected (mean score 2.79 ± 1.41). The services related to non-deferrable diseases were maintained in 22 UU.OO. (91.7%; 83.3% “yes” and 8.3% “enough”, mean score 3.63 ± 0.99).
7. Procedures and recommendations for healthcare professionals/users: In most of the UU.OO., the measures adopted were respected by both patients (100%; 54.2% “yes” and 45.8% “enough”) and relatives (95.8%; 62.5% “yes”, 33.3% “enough” and 4.2% “not enough”); as for the healthcare professionals, the recommendations were visible and clear in 91.7% of the UU.OO. (87.5% “yes” and 4.2% “enough”, with only 4.2% “not applicable”; mean score 3.67 ± 0.99) and the measures adopted were respected in 87.5% of them (83.3% “yes” and 4.2% “enough”, with 8.3% “not applicable”; mean score 3.50 ± 1.22).
8. Training, information, and management of health workers in the pandemic era: In 22 UU. OO. (91.7%; 87.5% “yes” and 4.2% “enough”), specific training was carried out to ensure the correct adoption of PPE (mean score 3.63 ± 1.11), and the health personnel working within the CPs were monitored with screening tests for SARS-CoV-2 (87.5% “yes”, 8.3% “enough” and 4.2% “not applicable”; mean score 3.75 ± 0.83), and kept as safe as possible through the use of PPE modulated on the basis of the different risk exposure (79.2% “yes”, 16.7% “enough” and 4.2% “not applicable”; mean score 3.67 ± 0.85).

## 4. Discussion

Several surveys have been proposed since the spread of the SARS-CoV-2 pandemic to assess the impact of the coronavirus on HCS [21,22,23,24,25], but none investigate the organizational and managerial responses of CPs during both the first and the second waves of the pandemic event.

CPs are often developed at a local level to meet specific needs; therefore, a well-designed care pathway includes a framework for the evaluation and assessment of its own effectiveness [26]. The main problems during the pandemic have been the need to reduce the outpatient visits number, have multidisciplinary meetings between physicians and not increase the work of healthcare workers directly involved in facing the emergency [27]. Nevertheless, all the analyzed CPs showed levels of performance ranging from Acceptable to Good, for both the organizational and management point of view (Table 4).

In particular, the management of cardiologic and diabetic patients has been simplified by remote monitoring techniques for precise parameters that can be recorded, stored, and remotely transmitted to the physician, facilitating the appropriate clinical decisions [28]. In fact, the cardiology and diabetic wards included in the CP ensured patients follow-up by implementing telemedicine, when possible, with varying results. Both doctors and patients welcomed this new approach, leading to conclude that its use will also continue in the future.

The COVID-19 pandemic has also led to a better organization of clinical activities and regular testing among healthcare workers, with better chances to grant patients’ protection, underlining the need to develop new protocols for the CPs already available [29,30]. It is notable that a low level of performance were found for the item “Impact on the treatment of patients also SARS-CoV-2 infected in the CPs”, that with a mean score 0.79 ± 0.22, was considered Not Acceptable.

During the first wave, the fear of contagion by entering the hospital or the outpatient clinics led to a reduction in the number of patients willing to start the treatment in three of the investigated CPs; this finding is consistent with those reported in other studies, some of which highlighted a reduction in new accesses to HCS [12,28,29,31].

Only during the second wave was there a positive stabilization of this trend. Nevertheless, the majority of the canceled visits were rescheduled in order to guarantee both the starting of new therapies and continuity of care for the patients already included in the CPs.

Oncologic and diabetic patients included in a CP and also infected by SARS-CoV-2 were admitted to the COVID ward of the same hospital, ensuring a partial continuity of care, as well as already reported for oncological care in other experiences and where specific CPs for COVID-19 were ensured [5,32]. Whereas, regarding the continuity of care for patients affected by both DSA and SARS-CoV-2, the number of cases was small, and it was not necessary to create separate pathways and/or wards. This is probably due to the fact that patients with a severe psychiatric illness already lead their life in social isolation, with reduced interactions on the job (when they have one) and very few family connections.

The structural and organizational changes necessary to keep working during the pandemic were specific for each HCS included in the survey, but the common goal was to increase social distancing in order to reduce the risk of contagion. The organization remained substantially unchanged, even if the different gradation of the positive answers suggests that a few structures managed to introduce only minimal changes compared to others.

One of the weaknesses highlighted by our survey is that in the majority of the CPs, the use of telemedicine was lower than expected. This is in contrast to the provisions of Lazio Region plan for reorganization, requalification, and development of the Regional Health Service 2019–2021 [33], which foresaw the use of telemedicine as a support in all clinical processes in order to maximize the efficiency and allowing a better interaction between the different care settings. Nevertheless, it has been shown that, even with the extra motivation linked to the pandemic, the majority of the UU.OO. did not properly introduce the necessary technological changes. The lack of successful implementation into service settings of telemedicine was also recorded in other studies [30,31], particularly within youth mental health care, oncology departments, and cardiology wards. On the contrary, the University of Melbourne registered a high interest in using telemedicine as part of care, also beyond the pandemic, in both patients and physicians [31].

However, it must be taken into account that in order to replace frontal visits with telemedicine, efficient and sure platforms must be available [30], also considering that some issues related to data privacy are yet to be solved [28,34].

In regard to the multidisciplinary meetings for the discussion of cases using teleconferencing, this new system was used both by our respondents and in other settings [12,29,32]. Some studies report additional information, for example, the study by Fersia et al. [29] was also focused on extra benefits ensured to patients’ care, as the teleconference systems allowed the involvement of other colleagues from different hospitals [29].

The results regarding the high level of healthcare workers informed about the vaccination campaign are encouraging (Table 3). This supports the importance of the information strategy that has accompanied the COVID-19 immunization campaign in Italy [35].

The results regarding the heart failure CP were similar to those found by Fersia et al. [29], even if this study was not specifically targeted on CPs. It was reported that all cardiology services (e.g., outpatient clinics, community services, and cardiac rehabilitation) sustained significant reductions and that telephone and video consultation services were adopted to minimize exposure risks to patients and staff [29].

The hereditary breast-ovarian cancers CP followed the general trend of modifying treatments to minimize potential exposure of vulnerable patients to SARS-CoV-2 and to reduce the risk during surgery or radiation therapy, which is consistent with the findings of other studies [12]. Other studies also highlighted that the continuity of oncological care was in any case guaranteed thanks to the use of protective devices, pre-triage of patients accessing the hospital, delay of non-urgent visits, and use of telemedicine for patients’ follow-up, in addition to periodical rhino-pharyngeal swabs for SARS-CoV-2 testing in healthcare workers [12,30,32]. In some cases, the surgical activities were carried out as outpatient services, therefore registering an increased activity in that area [32].

It is notable that, in our experience, the Psychoncology services gave support to all oncologic patients, regardless of the eventual COVID-19 infection, giving help and support by teleconsultation, not only to the patients but also to their families during the whole treatment period. At the same time, all kinds of organizational solutions in order to reschedule the canceled visits were implemented by introducing two parallel pathways: the regular CP for non-COVID patients and the ICP (integrated care pathway) for adult, non-pregnant SARS-CoV-2 positive patients [5].

It is interesting that the hereditary breast-ovarian cancers’ CP maintained a Good level of performance in all explored sections, with the exclusion of the “Impact on the treatment of patients also SARS-CoV-2 infected in the Clinical Pathway (Table 4).

As for the Diabetes CP, it maintained an acceptable level of performance and was a useful tool for the multidisciplinary care management of diabetic patients, particularly important as it has been supposed that social distancing, quarantine, and lockdown may have led to worsening of glucose control, also because of a decreased physical activity and an increased tendency to follow an unhealthy diet and lifestyle [28,36,37,38]. In this case, the great implementation of telemedicine (also in the form of remote monitoring) may have reduced diabetic complications [28].

The trend of the autism spectrum disorders (DSA) CP was the opposite of the other CPs; in fact, it showed an increase in both the request of services and in the number of patients willing to start the treatment during the pandemic. These findings are in line with those registered by the University of Melbourne, where the visits cancellation rate was low [31]. Half of our respondents declared to be using telemedicine satisfactorily in almost all conditions, such as in other experiences [31,34].

The authors are aware of some limits. Firstly, our data are related to the experience in one region and not extendible to a national level. With regard to this issue, Bosa et al. [39] reported that the Italian National Health System did not approach the pandemic as a united front, as differences were underlined from one region to another. In fact, misunderstandings and consequent tensions between central government and regions most likely lead some regions to take autonomous decisions (centrifugal drive), whereas others followed the government to avoid taking the burden of owning the responsibility (centripetal drive) [39]. Secondly, the different specific issues were not investigated in depth in order to avoid an excessive length of the questionnaire; this could have hidden important information, e.g., those related to the use of telematic monitoring of glucose in diabetic patients. Finally, at the moment, there are very few studies analyzing the impact of COVID-19 on specific CPs; therefore, it was difficult to compare our results to other experiences.

## 5. Conclusions

In conclusion, our results underline that CPs, notwithstanding the exceptional COVID-19 emergency and its impact on the different HCS settings, can be considered as resilience tools for patients’ care.

However, even considering their admirable results in facing the pandemic, CPs are disease-oriented clinical governance tools, and find their roots in the need for the care management of patients with a specific disease in specific settings; on the contrary, during the pandemic, our study demonstrates that patients with non-COVID-related illnesses, but SARS-CoV-2 positive, did not follow the specific CP but were treated within the COVID wards.

In light of this evidence, some changes are necessary to face any future challenges, such as pandemic emergencies. It is necessary to reinforce the integrated diagnostic-therapeutic systems of CPs, also considering their role in reducing the length of stay in hospital settings [40]. In this context, the rapid adoption of telemedicine can have an important impact on assistance and should be favored.

Moreover, it seems necessary to raise patients’ awareness about the need to start any kind of treatment as early as possible

## Figures and Tables

**Table 1:** Operational Units belonging to the Local Health Authorities (ASL)/Hospitals (AO) of the Lazio Region (Central Italy) who responded to the Survey; UOC: Complex Operational Unit; UOD: Departmental Operational Unit; UOS: Simple Operational Unit; OBI: (*Osservazione Breve Intensiva*) Intensive Brief Observation; DH: Day Hospital; PLS (*Pediatra di Libera Scelta*): General Paediatrician; UCP (*Unità di Cure Primarie*): Primary Care Unit.

Clinical Pathway	UU OO	ASL/AO
Heart Failure	UOC Cardiology	AO San Camillo, Rome (RM3)
	UOD Radiologic Emergency/Urgency	
	UOC Emergency Medicine, Emergency Department and OBI	
	UOSD Cardiology Integrated Services	
	UOD Shock and Trauma	
Hereditary breast-ovarian cancer	UOC Medical Genetics	University Hospital Sant’Andrea, Rome (RM1)
	UOC Oncology	
	UOS Diagnostic and Therapeutic Breast Unit (UDTS)	
	UOS Breast Radiology	
	UOC Gynecology	
	UOD Breast Surgery	
	UOD Psychoncology	
Autism Spectrum Disorders (ASD)	UOC Mental Health Center H1-H3	ASL RM6, Department of Mental Health and Pathological Addictions (DSM-DP)
	UOC Mental Health Center H4-H6	
	UOC Protection of Mental Health and Rehabilitation of the Age of Development (TMSREE)	
	Psychiatric Service of Diagnosis and Cure	
	DH Psychiatric	
	PLS	
Diabetes	UOC District C Management-Atina Community Health Center (*Casa Della Salute*)	ASL Frosinone-District C and D
	UOS Primary Care (*Assistenza Sanitaria di Base*, ASB) District C and D	
	UOD Endocrinology and Metabolic Diseases	
	UOC Public Relations Office-Single Access Point (*Punto Unico di Accesso*, PUA)	
	UCP of General Practitioners—Atina Health Center	
	Outpatient specialistic visits in Cardiology, Diabetology, and Ophthalmology	

**Table 2:** Impact of COVID-19 pandemic on clinical pathways; data analysis of the eight sections of the survey (37 items) using 4-point Likert scale: yes = 4, enough = 3, not enough = 2, not at all = 1, not applicable = 0. Means and standard deviations (SDs) were calculated for each section of the survey, overall and for each CP. HF: heart failure; HBOC: hereditary breast-ovarian cancers; ASD: autism spectrum disorders; D: diabetes.

	COVID Survey Sections	Clinical Pathways (CPs)					Level of Performance *
		Overall (2.86 ± 0.08)	HF (2.54 ± 0.17)	HBOC (3.19 ± 0.12)	ASD (2.81 ± 0.14)	D 2.90 ± 0.25	Overall: Acceptable
1	Context analysis	2.63 ± 0.20	2.40 ± 0.34	3.00 ± 0.25	2.88 ± 0.34	2.25 ± 0.61	HBOC: Good Overall, ASD, HF, D: Acceptable
2	Patients access to CP/UO	3.68 ± 0.23	3.20 ± 0.33	3.62 ± 0.34	3.89 ± 0.10	4.00 ± 0.82	Good
3	Impact on the treatment of NON-COVID patients in the clinical pathway	2.80 ± 0.26	2.27 ± 0.59	3.43 ± 0.32	2.61 ± 0.48	2.89 ± 0.69	HBOC: Good Overall, ASD, HF, D: Acceptable
4	Impact on the treatment of patients also SARS-CoV-2 infected in the Clinical Pathway	0.79 ± 0.22	0.88 ± 0.63	0.83 ± 0.41	0.77 ± 0.38	0.67 ± 0.33	Not Acceptable
5	Impact of the COVID-19 pandemic on patient management	3.09 ± 0.28	2.84 ± 0.58	3.34 ± 0.49	3.27 ± 0.45	2.90 ± 0.70	Overall, HBOC, ASD: Good HF, D: Acceptable
6	Structural and organizational changes of the CP/UO	3.10 ± 0.26	2.37 ± 0.59	3.64 ± 0.26	3.19 ± 0.39	3.19 ± 0.73	Overall, HBOC, ASD, D: Good HF: Acceptable
7	Procedures and recommendations for healthcare professionals/users	3.63 ± 0.22	3.20 ± 0.24	3.81 ± 0.21	3.53 ± 0.27	3.97 ± 0.81	Good
8	Training, information and management of health workers in the pandemic era	3.19 ± 0.26	3.20 ± 0.47	3.88 ± 0.22	2.36 ± 0.56	3.31 ± 0.74	Overall, HBOC, HF, D: Good ASD: Acceptable

* cut off for acceptable level of performance of the CP mean score > 1.80 and cut off for good level of performance of the CP mean score > 2.99; a mean score < 1.80 was considered as a not acceptable level of performance.

**Table 3:** The questionnaire consists of eight sections with 37 items, and each one gives, as possible answers, five different verbal categories connected to a percentage range (specifically: yes ≥ 75%; 51% ≤ enough ≤ 74%; 26% ≤ not enough ≤ 50%; and not at all ≤ 25%), and respondents are asked to indicate the verbal category that comes closest to their position.

	Yes *n* (%)	Enough *n* (%)	Not Enough *n* (%)	Not at All *n* (%)	NA *n* (%)	Total Score	Mean Score ± DS	Level of Performance *
1. Contex Analysis							2.63 ± 0.20	Acceptable
During the COVID-19 pandemic, did patients accept treatment despite the fear of contagion?	7 (29.2)	8 (33.3)	2 (8.3)	5 (20.8)	2 (8.3)	61	2.54 ± 1.32	Acceptable
Compared to the same period of the previous year, during the first wave of the pandemic, did the number of accesses remain stable?	2 (8.3)	10 (41.7)	3 (12.5)	9 (37.5)	0 (0.0)	53	2.21 ± 1.04	Acceptable
Compared to the first wave of the pandemic, did the number of patients undertaking the care pathways remained stable during the second wave?	15 (62.5)	5 (20.8)	3 (12.5)	0 (0.0)	1 (4.2)	81	3.38 ± 0.99	Good
Has the volume of procedures remained stable during first and second waves compared to the same period of the previous year?	3 (12.5)	13 (54.2)	3 (12.5)	3 (12.5)	2 (8.3)	60	2.50 ± 1.12	Acceptable
2. Patients access to CP/UO							3.68 ± 0.23	Good
Do you use a pre-triage module during treatment?	20 (83.3)	0 (0.0)	0 (0.0)	3 (12.5)	1 (4.2)	83	3.46 ± 1.22	Good
Are security measures taken?	22 (91.7)	2 (8.3)	0 (0.0)	0 (0.0)	0 (0.0)	94	3.92 ± 0.28	Good
Are social distancing measures being taken?	19 (79.2)	4 (16.7)	0 (0.0)	1 (4.2)	0 (0.0)	89	3.71 ± 0.68	Good
3. Impact on the treatment of NON-COVID patients in the Clinical Pathway							2.80 ± 0.26	Acceptable
Was the start of the treatment within the care pathway guaranteed to the patients anyway?	16 (66.7)	5 (20.8)	1 (4.2)	1 (4.2)	1 (4.2)	82	3.42 ± 1.04	Good
Have the cancelled visits been rescheduled and recovered?	14 (58.3)	4 (16.7)	0 (0.0)	1 (4.2)	5 (20.8)	69	2.88 ± 1.62	Acceptable
Has remote monitoring been activated for patients who could not interrupt the treatment (telemedicine)?	4 (16.7)	10 (41.7)	3 (12.5)	2 (8.3)	5 (20.8)	54	2.25 ± 1.39	Acceptable
4. Impact on the treatment of patients also SARS-CoV-2 infected in the Clinical Pathway							0.79 ± 0.22	Not Acceptable
Have you treated patients affected by COVID-19 within the care pathway/hospital ward?	0 (0.0)	2 (8.3)	3 (12.5)	13 (54.2)	6 (25.0)	25	1.04 ± 0.84	Not Acceptable
If yes or enough: in hospital in COVID wards?	0 (0.0)	0 (0.0)	1 (4.2)	3 (12.5)	20 (83.3)	5	0.21 ± 0.50	Not Acceptable
If yes or enough: was it in COVID wards with telephone counselling?	0 (0.0)	0 (0.0)	2 (8.3)	3 (12.5)	19 (79.2)	7	0.29 ± 0.61	Not Acceptable
Have the care pathways for COVID and NON_COVID patients been separated?	12 (50.0)	1 (4.2)	0 (0.0)	2 (8.3)	9 (37.5)	53	2.21 ± 1.89	Acceptable
5. Impact of the COVID-19 pandemic on patient management							3.09 ± 0.28	Good
Have *ad hoc* organizational solutions been implemented for patient management compared to the pre-pandemic era?	21 (87.5)	2 (8.3)	0 (0.0)	1 (4.2)	0 (0.0)	91	3.79 ± 0.64	Good
Has therapeutic continuity been ensured within the pathway care?	20 (83.3)	4 (16.7)	0 (0.0)	0 (0.0)	0 (0.0)	92	3.83 ± 0.37	Good
Have technological solutions, such as telemedicine, been adopted for patient follow-up?	5 (20.8)	8 (33.3)	4 (16.7)	2 (8.3)	5 (20.8)	54	2.25 ± 1.42	Acceptable
Did all hospitalized patients repeat the screening test for SARS-CoV-2 several times during the hospitalization period?	11 (45.8)	0 (0.0)	1 (4.2)	1 (4.2)	11 (45.8)	47	1.96 ± 1.93	Acceptable
Has the correct use of PPE (personal protective equipment) by healthcare professionals and patients been monitored?	22 (91.7)	0 (0.0)	0 (0.0)	1 (4.2)	1 (4.2)	89	3.71 ± 0.98	Good
6. Structural and organizational changes of the CP/UO							3.10 ± 0.26	Good
Has the care pathway/hospital ward remained unchanged from an organizational point of view?	10 (41.7)	9 (37.5)	2 (8.3)	1 (4.2)	2 (8.3)	72	2.77 ± 1.40	Acceptable
Were outpatient and/or surgical activities guaranteed?	14 (58.3)	6 (25.0)	0 (0.0)	1 (4.2)	3 (12.5)	75	3.13 ± 1.36	Good
Has the timing of the transition of a patient from one care setting to another within the care pathway/hospital ward been respected?	9 (37.5)	10 (41.7)	0 (0.0)	1 (4.2)	4 (16.7)	67	2.79 ± 1.41	Acceptable
Have there been multidisciplinary discussions about the patients’ health conditions?	12 (50.0)	6 (25.5)	2 (8.3)	0 (0.0)	4 (16.7)	70	2.92 ± 1.44	Acceptable
Have structural changes been made to encourage social distancing?	17 (70.8)	4 (16.7)	1 (4.2)	1 (4.2)	1 (4.2)	83	3.46 ± 1.04	Good
Have the services relating to non-deferrable diseases been guaranteed?	20 (83.3)	2 (8.3)	0 (0.0)	1 (4.2)	1 (4.2)	87	3.63 ± 0.99	Good
7. Procedures and recommendations for healthcare professionals/users							3.63 ± 0.22	Good
Have recommendations for the patients been made clear and visible?	21 (87.5)	2 (8.3)	0 (0.0)	1 (4.2)	0 (0.0)	91	3.79 ± 0.64	Good
If yes, or enough, have they been respected?	13 (54.2)	11 (45.8)	0 (0.0)	0 (0.0)	0 (0.0)	85	3.54 ± 0.50	Good
Have recommendations for relatives been made clear and visible?	22 (91.7)	1 (4.2)	0 (0.0)	1 (4.2)	0 (0.0)	92	3.83 ± 0.62	Good
If yes, or enough, have they been respected?	15 (62.5)	8 (33.3)	1 (4.2)	0 (0.0)	0 (0.0)	86	3.58 ± 0.57	Good
Have recommendations for healthcare professionals been made clear and visible?	21 (87.5)	1 (4.2)	0 (0.0)	1 (4.2)	1 (4.2)	88	3.67 ± 0.99	Good
If yes, or enough, have they been respected?	20 (83.3)	1 (4.2)	0 (0.0)	1 (4.2)	2 (8.3)	84	3.50 ± 1.22	Good
8. Training, information and management of health workers in the pandemic era							3.19 ± 0.26	Good
Have health care workers involved in care pathways/hospital wards been trained on the dressing-doffing PPE procedures?	21 (87.5)	1 (4.2)	0 (0.0)	0 (0.0)	2 (8.3)	87	3.63 ± 1.11	Good
Has the exposed health care personnel been periodically subjected to rhino-pharyngeal swabs to evaluate the possible positivity for SARS-CoV-2?	21 (87.5)	2 (8.3)	0 (0.0)	0 (0.0)	1 (4.2)	90	3.75 ± 0.83	Good
Has the staff been equipped with PPE of modulated efficiency with respect to the professional risk to which they have been exposed?	19 (79.2)	4 (16.7)	0 (0.0)	0 (0.0)	1 (4.2)	88	3.67 ± 0.85	Good
Have dirty paths and clean access paths to clinical departments been organized?	10 (41.7)	3 (12.5)	0 (0.0)	0 (0.0)	11 (45.8)	49	2.04 ± 1.90	Acceptable
Has the corporate anti-COVID vaccination program been performed using the employee booking portal?	16 (66.7)	0 (0.0)	0 (0.0)	3 (12.5)	5 (20.8)	67	2.79 ± 1.73	Acceptable
In the company/facility, was the anti-COVID19 vaccination campaign preceded by an information campaign on the technical characteristics, methods of setting up and administering the vaccine?	20 (83.3)	0 (0.0)	0 (0.0)	2 (8.3)	2 (8.3)	82	3.42 ± 1.32	Good

* cut off for acceptable level of performance of the CP mean score > 1.80 and cut off for good level of performance of the CP mean score > 2.99; a mean score < 1.80 was considered as a not acceptable level of performance.

**Table 4:** Total results regarding every CP.

Clinical Pathway	COVID Survey Section	Total Score	Mean Score ± DS	Level of Performance *
Heart Failure		486	2.54 ± 0.17	Acceptable
	Context analysis	48	2.40 ± 0.34	Acceptable
	Patients access to CP/UO	48	3.20 ± 0.33	Good
	Impact on the treatment of non-COVID patients in the clinical pathway	34	2.27 ± 0.59	Acceptable
	Impact on the treatment of patients also SARS-CoV-2 infected in the Clinical Pathway	22	0.88 ± 0.63	Not Acceptable
	Impact of the COVID-19 pandemic on patient management	71	2.84 ± 0.58	Acceptable
	Structural and organizational changes of the CP/UO	71	2.37 ± 0.59	Acceptable
	Procedures and recommendations for healthcare professionals/users	96	3.20 ± 0.24	Good
	Training, information, and management of health workers in the pandemic era	96	3.20 ± 0.47	Good
Hereditary Breast-ovarian Cancer		852	3.19 ± 0.12	Good
	Context analysis	84	3.00 ± 0.25	Good
	Patients access to CP/UO	76	3.62 ± 0.34	Good
	Impact on the treatment of non-COVID patients in the clinical pathway	72	3.43 ± 0.32	Good
	Impact on the treatment of patients also SARS-CoV-2 infected in the Clinical Pathway	29	0.83 ± 0.41	Not Acceptable
	Impact of the COVID-19 pandemic on patient management	117	3.34 ± 0.49	Good
	Structural and organizational changes of the CP/UO	153	3.64 ± 0.26	Good
	Procedures and recommendations for healthcare professionals/users	160	3.81 ± 0.21	Good
	Training, information, and management of health workers in the pandemic era	163	3.88 ± 0.22	Good
Diabetes		660	2.90 ± 0.25	Acceptable
	Context analysis	54	2.25 ± 0.61	Acceptable
	Patients access to CP/UO	72	4.00 ± 0.82	Good
	Impact on the treatment of non-COVID patients in the clinical pathway	52	2.89 ± 0.69	Acceptable
	Impact on the treatment of patients also SARS-CoV-2 infected in the Clinical Pathway	20	0.67 ± 0.33	Not Acceptable
	Impact of the COVID-19 pandemic on patient management	87	2.90 ± 0.70	Acceptable
	Structural and organizational changes of the CP/UO	115	3.19 ± 0.73	Good
	Procedures and recommendations for healthcare professionals/users	143	3.97 ± 0.81	Good
	Training, information and management of health workers in the pandemic era	119	3.31 ± 0.74	Good
Autism Spectrum Disorders		634	2.81 ± 0.14	Acceptable
	Context analysis	69	2.88 ± 0.34	Acceptable
	Patients access to CP/UO	70	3.89 ± 0.10	Good
	Impact on the treatment of non-COVID patients in the clinical pathway	47	2.61 ± 0.48	Acceptable
	Impact on the treatment of patients also SARS-CoV-2 infected in the Clinical Pathway	23	0.77 ± 0.38	Not Acceptable
	Impact of the COVID-19 pandemic on patient management	98	3.27 ± 0.45	Good
	Structural and organizational changes of the CP/UO	115	3.19 ± 0.39	Good
	Procedures and recommendations for healthcare professionals/users	127	3.53 ± 0.27	Good
	Training, information, and management of health workers in the pandemic era	85	2.36 ± 0.56	Acceptable

* cut off for acceptable level of performance of the CP mean score > 1.80 and cut off for good level of performance of the CP mean score > 2.99; a mean score < 1.80 was considered as a not acceptable level of performance.

## Data Availability

All relevant data can be found in the Supplementary Materials.

## Supplementary Materials

The following are available online at https://www.mdpi.com/article/10.3390/ijerph19020635/s1, Table S1: COVID SURVEY; Table S2: Clinical Pathways’ data; Supplementary Material SM1: Statistical analysis.
