# Supplementary material for: Impact of the COVID-19 Pandemic on Clinical Pathways for Non-SARS-CoV-2 Related Diseases in the Lazio Region, Italy
Source: Int J Environ Res Public Health. 2022 Jan 6;19(2):635. doi: 10.3390/ijerph19020635 (PMC8776184; doi:10.3390/ijerph19020635)
Supplement: Supplementary file 1 [file ijerph-19-00635-s001.zip › SUPPLEMENTARY Material S1.pdf]

SM1 Statistical analysis

Total score is calculated from the sum of the Likert scale values, where "yes" is equal to 4, "enough" to 3, "not enough" to 2, "not at all" to 1 and “not applicable” to 0.

CONTEXT ANALYSIS

1. During the COVID-19 pandemic, did patients accept treatment despite the fear of contagion?

Answer choices	*Responses (%)*	*Responses (number)*	*Responses (score)*
Yes	29.2%	7	7*4=28
Enough	33.3%	8	8*3=24
Not enough	8.3%	2	2*2=4
Not at all	20.8%	5	5*1=5
Not applicable	8.3%	2	2*0=0

Answered	*Total score*	*Mean*	*Variance*	*SD*
24	61	2.54	1.75	1.32

2. Compared to the same period of the previous year, during the first wave of the pandemic, did the number of accesses remain stable?

Answer choices	*Responses (%)*	*Responses (number)*	*Responses (score)*
Yes	8.3%	2	2*4=8
Enough	41.7%	10	10*3=30
Not enough	12.5%	3	3*2=6
Not at all	37.5%	9	9*1=9
Not applicable	0%	0	0*0=0

Answered	*Total score*	*Mean*	*Variance*	*SD*
24	53	2.21	1.08	1.04

3. Compared with the the first wave of the pandemic, did the number of patients undertaking the care pathways remained stable during the second wave?

Answer choices	*Responses (%)*	*Responses (number)*	*Responses (score)*
Yes	62.5%	15	15*4=60
Enough	20.8%	5	5*3=15
Not enough	12.5%	3	3*2=6
Not at all	0%	0	0*1=0
Not applicable	4.2%	1	1*0=0

Answered	*Total score*	*Mean*	*Variance*	*SD*
24	81	3.38	0.98	0.99

4. Has the volume of procedures remained stable during first and second waves compared to the same period of the previous year?

Answer choices	Responses (%)	Responses (number)	Responses (score)
Yes	12.5%	3	3*4=12
Enough	54.2%	13	13*3=39
Not enough	12.5%	3	3*2=6
Not at all	12.5%	3	3*1=3
Not applicable	8.3%	2	2*0=0

Answered	Total score	Mean	Variance	SD
24	60	2.50	1.25	1.12

PATIENTS ACCESS TO CP/ UO

5. Do you use a pre-triage module during treatment?

Answer choices	Responses (%)	Responses (number)	Responses (score)
Yes	83.3%	20	20*4=80
Enough	0.0%	0	0*3=0
Not enough	0.0%	0	0*2=0
Not at all	12.5%	3	3*1=3
Not applicable	4.2%	1	1*0=0

Answered	Total score	Mean	Variance	SD
24	83	3.46	1.50	1.22

6. Are security measures being taken?

Answer choices	Responses (%)	Responses (number)	Responses (score)
Yes	91.7%	22	22*4=88
Enough	8.3%	2	2*3=6
Not enough	0.0%	0	0*2=0
Not at all	0.0%	0	0*1=0
Not applicable	0.0%	0	0*0=0

Answered	Total score	Mean	Variance	SD
24	94	3.92	0.08	0.28

7. Are social distancing measures being taken?

Answer choices	Responses (%)	Responses (number)	Responses (score)
Yes	79.2%	19	19*4=76
Enough	16.7%	4	4*3=12
Not enough	0.0%	0	0*2=0
Not at all	4.2%	1	1*1=1
Not applicable	0.0%	0	0*0=0

Answered	Total score	Mean	Variance	SD
24	89	3.71	0.46	0.68

IMPACT ON ACCESS TO CPs FOR NO-COVID PATIENTS

8. Was the start of the treatment within the care pathway garanteed to the patients anyway?

Answer choices	*Responses (%)*	*Responses (number)*	*Responses (score)*
Yes	66.7%	16	16*4=64
Enough	20.8%	5	5*3=15
Not enough	4.2%	1	1*2=2
Not at all	4.2%	1	1*1=1
Not applicable	4.2%	1	1*0=0

Answered	*Total score*	*Mean*	*Variance*	*SD*
24	82	3.42	1.08	1.04

9. Have the cancelled visits been rescheduled and recovered?

Answer choices	*Responses (%)*	*Responses (number)*	*Responses (score)*
Yes	58.3%	14	14*4=56
Enough	16.7%	4	4*3=12
Not enough	0.0%	0	0*2=0
Not at all	4.2%	1	1*1=1
Not applicable	20.8%	5	5*0=0

Answered	*Total score*	*Mean*	*Variance*	*SD*
24	69	2.88	2.61	1.62

10. Has remote monitoring been activated for patients who could not interrupt the treatment (telemedicine)?

Answer choices	*Responses (%)*	*Responses (number)*	*Responses (score)*
Yes	16.7%	4	4*4=16
Enough	41.7%	10	10*3=30
Not enough	12.5%	3	3*2=6
Not at all	8.3%	2	2*1=2
Not applicable	20.8%	5	5*0=0

Answered	*Total score*	*Mean*	*Variance*	*SD*
24	54	2.25	1.94	1.39

IMPACT ON ACCESS TO CPs FOR PATIENTS ALSO SARS COV-2 INFECTED

11. Have you treated patients affected by COVID-19 within the care pathway/hospital ward?

Answer choices	*Responses (%)*	*Responses (number)*	*Responses (score)*
Yes	0.0%	0	0*4=0
Enough	8.3%	2	2*3=6
Not enough	12.5%	3	3*2=6
Not at all	54.2%	13	13*1=13
Not applicable	25.0%	6	0*0=0

Answered	*Total score*	*Mean*	*Variance*	*SD*
24	25	1.04	0.71	0.84

12. If yes or enough: was it inside the hospital in COVID wards?

Answer choices	*Responses (%)*	*Responses (number)*	*Responses (score)*
Yes	0.0%	0	0*4=0
Enough	0.0%	0	0*3=0
Not enough	4.2%	1	1*2=2
Not at all	12.5%	3	3*1=3
Not applicable	83.3%	20	20*0=0

Answered	*Total score*	*Mean*	*Variance*	*SD*
24	5	0.21	0.25	0.50

13. If yes or enough: was it in COVID wards with telephone counselling?

Answer choices	*Responses (%)*	*Responses (number)*	*Responses (score)*
Yes	0.0%	0	0*4=0
Enough	0.0%	0	0*3=0
Not enough	8.3%	2	2*2=4
Not at all	12.5%	3	3*1=3
Not applicable	79.2%	19	19*0=0

Answered	*Total score*	*Mean*	*Variance*	*SD*
24	7	0.29	0.37	0.61

14. Have the care pathways for COVID and NON_COVID patients been separated?

Answer choices	*Responses (%)*	*Responses (number)*	*Responses (score)*
Yes	50.0%	12	12*4=48
Enough	4.2%	1	1*3=3
Not enough	0.0%	0	0*2=0
Not at all	8.3%	2	2*1=2
Not applicable	37.5%	9	9*0=0

Answered	*Total score*	*Mean*	*Variance*	*SD*
24	53	2.21	3.58	1.89

IMPACT OF THE COVID-19 PANDEMIC ON PATIENT MANAGEMENT

15. Have ad hoc organizational solutions been implemented for patient management compared to the pre-pandemic era?

Answer choices	*Responses (%)*	*Responses (number)*	*Responses (score)*
Yes	87.5%	21	21*4=84
Enough	8.3%	2	2*3=6
Not enough	0.0%	0	0*2=0
Not at all	4.2%	1	1*1=1
Not applicable	0.0%	0	0*0=0

Answered	*Total score*	*Mean*	*Variance*	*SD*
24	91	3.79	0.41	0.64

16. Has therapeutic continuity been ensured within the pathway care?

Answer choices	*Responses (%)*	*Responses (number)*	*Responses (score)*
Yes	83.3%	20	20*4=80
Enough	16.7%	4	4*3=12
Not enough	0.0%	0	0*2=0
Not at all	0.0%	0	0*1=0
Not applicable	0.0%	0	0*0=0

Answered	*Total score*	*Mean*	*Variance*	*SD*
24	92	3.83	0.14	0.37

17. Have technological solutions, such as telemedicine, been adopted for patient follow-up?

Answer choices	*Responses (%)*	*Responses (number)*	*Responses (score)*
Yes	20.8%	5	5*4=20
Enough	33.3%	8	8*3=24
Not enough	16.7%	4	4*2=8
Not at all	8.3%	2	2*1=2
Not applicable	20.8%	5	5*0=0

Answered	*Total score*	*Mean*	*Variance*	*SD*
24	54	2.25	2.02	1.42

18. Did all hospitalized patients repeat the screening test for SARS-Cov-2 several times during the hospitalization period?

Answer choices	*Responses (%)*	*Responses (number)*	*Responses (score)*
Yes	45.8%	11	11*4=44
Enough	0.0%	0	0*3=0
Not enough	4.2%	1	1*2=2
Not at all	4.2%	1	1*1=1
Not applicable	45.8%	11	11*0=0

Answered	*Total score*	*Mean*	*Variance*	*SD*
24	47	1.96	3.71	1.93

19. Has the correct use of PPE (personal protective equipment) by healthcare professionals and patients been monitored?

Answer choices	*Responses (%)*	*Responses (number)*	*Responses (score)*
Yes	91.7%	22	22*4=88
Enough	0.0%	0	0*3=0
Not enough	0.0%	0	0*2=0
Not at all	4.2%	1	1*1=1
Not applicable	4.2%	1	1*0=0

Answered	*Total score*	*Mean*	*Variance*	*SD*
24	89	3.71	0.96	0.98

STRUCTURAL AND ORGANIZATIONAL CHANGES OF THE CP/UO

20. Has the care pathway/hospital ward remained unchanged from an organizational point of view?

Answer choices	*Responses (%)*	*Responses (number)*	*Responses (score)*
Yes	41.7%	10	10*4=40
Enough	37.5%	9	9*3=27
Not enough	8.3%	2	2*2=4
Not at all	4.2%	1	1*1=1
Not applicable	8.3%	2	2*0=0

Answered	*Total score*	*Mean*	*Variance*	*SD*
24	72	3.00	1.19	1.40

21. Were outpatient and/or surgical activities guaranteed?

Answer choices	*Responses (%)*	*Responses (number)*	*Responses (score)*
Yes	58.3%	14	14*4=56
Enough	25.0%	6	6*3=18
Not enough	0.0%	0	0*2=0
Not at all	4.2%	1	1*1=1
Not applicable	12.5%	3	3*0=0

Answered	Total score	Mean	Variance	SD
24	75	3.13	1.86	1.36

22. Has the timing of the transition of a patient from one care setting to another within the care pathway/hospital ward been respected?

Answer choices	*Responses (%)*	*Responses (number)*	*Responses (score)*
Yes	37.5%	9	9*4=36
Enough	41.7%	10	10*3=30
Not enough	0.0%	0	0*2=0
Not at all	4.2%	1	1*1=1
Not applicable	16.7%	4	4*0=0

Answered	Total score	Mean	Variance	SD
24	67	2.79	2.00	1.41

23. Have there been multidisciplinary discussions about the patients' health conditions?

Answer choices	*Responses (%)*	*Responses (number)*	*Responses (score)*
Yes	50.0%	12	12*4=48
Enough	25.0%	6	6*3=18
Not enough	8.3%	2	2*2=4
Not at all	0.0%	0	0*1=0
Not applicable	16.7%	4	4*0=0

Answered	Total score	Mean	Variance	SD
24	70	2.92	2.08	1.44

24. Have structural changes been made to encourage social distancing?

Answer choices	*Responses (%)*	*Responses (number)*	*Responses (score)*
Yes	70.8%	17	17*4=68
Enough	16.7%	4	4*3=12
Not enough	4.2%	1	1*2=2
Not at all	4.2%	1	1*1=1
Not applicable	4.2%	1	1*0=0

Answered	Total score	Mean	Variance	SD
24	83	3.46	1.08	1.04

25. Have the services relating to non-deferrable diseases been guaranteed?

Answer choices	*Responses (%)*	*Responses (number)*	*Responses (score)*
Yes	83.3%	20	20*4=80
Enough	8.3%	2	2*3=6
Not enough	0.0%	0	0*2=0
Not at all	4.2%	1	1*1=1
Not applicable	4.2%	1	1*0=0

Answered	*Total score*	*Mean*	*Variance*	*SD*
24	87	3.63	0.98	0.99

PROCEDURES AND RECOMMENDATIONS FOR HEALTHCARE PROFESSIONALS / USERS

26. Have recommendations for the patients been made clear and visible?

Answer choices	*Responses (%)*	*Responses (number)*	*Responses (score)*
Yes	87.5%	21	21*4=84
Enough	8.3%	2	2*3=6
Not enough	0.0%	0	0*2=0
Not at all	4.2%	1	1*1=1
Not applicable	0.0%	0	0*0=0

Answered	*Total score*	*Mean*	*Variance*	*SD*
24	91	3.79	0.41	0.64

27. If yes, or enough, have they been respected?

Answer choices	*Responses (%)*	*Responses (number)*	*Responses (score)*
Yes	54.2%	13	13*4=52
Enough	45.8%	11	11*3=33
Not enough	0.0%	0	0*2=0
Not at all	0.0%	0	0*1=0
Not applicable	0.0%	0	0*0=0

Answered	*Total score*	*Mean*	*Variance*	*SD*
24	85	3.54	0.25	0.50

28. Have recommendations for relatives been made clear and visible?

Answer choices	*Responses (%)*	*Responses (number)*	*Responses (score)*
Yes	91.7%	22	22*4=88
Enough	4.2%	1	1*3=3
Not enough	0.0%	0	0*2=0
Not at all	4.2%	1	1*1=1
Not applicable	0.0%	0	0*0=0

Answered	*Total score*	*Mean*	*Variance*	*SD*
24	92	3.83	0.39	0.62

29. If yes, or enough, have they been respected?

Answer choices	*Responses (%)*	*Responses (number)*	*Responses (score)*
Yes	62.5%	15	15*4=60
Enough	33.3%	8	8*3=24
Not enough	4.2%	1	1*2=2
Not at all	0.0%	0	0*1=0
Not applicable	0.0%	0	0*0=0

Answered	*Total score*	*Mean*	*Variance*	*SD*
24	86	3.58	0.33	0.57

30. Have recommendations for healthcare professionals been made clear and visible?

Answer choices	*Responses (%)*	*Responses (number)*	*Responses (score)*
Yes	87.5%	21	21*4=84
Enough	4.2%	1	1*3=3
Not enough	0.0%	0	0*2=0
Not at all	4.2%	1	1*1=1
Not applicable	4.2%	1	1*0=0

Answered	*Total score*	*Mean*	*Variance*	*SD*
24	88	3.67	0.97	0.99

31. If yes, or enough, have they been respected?

Answer choices	*Responses (%)*	*Responses (number)*	*Responses (score)*
Yes	83.3%	20	20*4=80
Enough	4.2%	1	1*3=3
Not enough	0.0%	0	0*2=0
Not at all	4.2%	1	1*1=1
Not applicable	8.3%	2	2*0=0

Answered	*Total score*	*Mean*	*Variance*	*SD*
24	84	3.50	1.50	1.22

TRAINING, INFORMATION AND MANAGEMENT OF HEALTH WORKERS IN THE PANDEMIC ERA

32. Have health care workers involved in care pathways/hospital wards been trained on the dressing-doffing PPE procedures?

Answer choices	Responses (%)	Responses (number)	Responses (score)
Yes	87.5%	21	21*4=84
Enough	4.2%	1	1*3=3
Not enough	0.0%	0	0*2=0
Not at all	0.0%	0	0*1=0
Not applicable	8.3%	2	2*0=0

Answered	Total score	Mean	Variance	SD
24	87	3.63	1.23	1.11

33. Has the exposed health care personnel been periodically subjected to rhino-pharyngeal swabs to evaluate the possible positivity for SARS-CoV-2?

Answer choices	Responses (%)	Responses (number)	Responses (score)
Yes	87.5%	21	21*4=84
Enough	8.3%	2	2*3=6
Not enough	0.0%	0	0*2=0
Not at all	0.0%	0	0*1=0
Not applicable	4.2%	1	1*0=0

Answered	Total score	Mean	Variance	SD
24	90	3.75	0.69	0.83

34. Has the staff been equipped with PPE of modulated efficiency with respect to the professional risk to which they have been exposed?

Answer choices	Responses (%)	Responses (number)	Responses (score)
Yes	79.2%	19	19*4=76
Enough	16.7%	4	4*3=12
Not enough	0.0%	0	0*2=0
Not at all	0.0%	0	0*1=0
Not applicable	4.2%	1	1*0=0

Answered	Total score	Mean	Variance	SD
24	88	3.67	0.72	0.85

35. Have dirty paths and clean access paths to clinical departments been organized?

Answer choices	Responses (%)	Responses (number)	Responses (score)
Yes	41.7%	10	10*4=40
Enough	12.5%	3	3*3=9
Not enough	0.0%	0	0*2=0
Not at all	0.0%	0	0*1=0
Not applicable	45.8%	11	11*0=0

Answered	Total score	Mean	Variance	SD
24	49	2.04	3.62	1.90

36. Has the corporate anti-COVID vaccination program been performed using the employee booking portal?

Answer choices	Responses (%)	Responses (number)	Responses (score)
Yes	66.7%	16	16*4=64
Enough	0.0%	0	0*3=0
Not enough	0.0%	0	0*2=0
Not at all	12.5%	3	3*1=3
Not applicable	20.8%	5	5*0=0

Answered	Total score	Mean	Variance	SD
24	67	2.79	3.00	1.73

37. In the company/facility, was the anti-COVID19 vaccination campaign preceded by an information campaign on the technical characteristics, methods of setting up and administering the vaccine?

Answer choices	Responses (%)	Responses (number)	Responses (score)
Yes	83.3%	20	20*4=80
Enough	0.0%	0	0*3=0
Not enough	0.0%	0	0*2=0
Not at all	8.3%	2	2*1=2
Not applicable	8.3%	2	2*0=0

Answered	Total score	Mean	Variance	SD
24	82	3.42	1.74	1.32

GENERAL DATA REGARDING EACH CP DIVIDED PER SECTION

Heart Failure CP

Total score	Mean	Variance	SD
486	2.54	0.03	0.17

1. Context analysis

Total score	Mean	Variance	SD
**48**	2.40	0.12	0.34

2. Patients access to care pathways/Operational Unit;

Total score	Mean	Variance	SD
**48**	3.20	0.11	0.33

3. Impact on the treatment of non-COVID patients in the CPs;

Total score	*Mean*	*Variance*	*SD*
**34**	2.27	0.35	0.59

4. Impact on the treatment of patients also SARS-COV-2 infected in the CPs;

Total score	*Mean*	*Variance*	*SD*
**22**	0.88	0.40	0.63

5. Impact of the COVID-19 pandemic on patient management;

Total score	*Mean*	*Variance*	*SD*
**71**	2.84	0.34	0.58

6. Structural and organizational changes of the CP/UO;

Total score	*Mean*	*Variance*	*SD*
**71**	2.37	0.34	0.59

7. Procedures and recommendations for healthcare professionals / users;

Total score	*Mean*	*Variance*	*SD*
**96**	3.20	0.06	0.24

8. Training, information, and management of health workers in the pandemic era.

Total score	*Mean*	*Variance*	*SD*
**96**	3.20	0.22	0.47

Hereditary Breast-Ovarian Cancers CP

Total score	*Mean*	*Variance*	*SD*
852	***3.19***	***0.01***	***0.12***

1. Context analysis

Total score	*Mean*	*Variance*	*SD*
**84**	3.00	0.06	0.25

2. Patients access to care pathways/Operational Unit;

Total score	*Mean*	*Variance*	*SD*
**76**	3.62	0.12	0.34

3. Impact on the treatment of non-COVID patients in the CPs;

Total score	*Mean*	*Variance*	*SD*
**72**	3.43	0.10	0.32

4. Impact on the treatment of patients also SARS-COV-2 infected in the CPs;

Total score	*Mean*	*Variance*	*SD*
**29**	0.83	0.17	0.41

5. Impact of the COVID-19 pandemic on patient management;

Total score	*Mean*	*Variance*	*SD*
**117**	3.34	0.24	0.49

6. Structural and organizational changes of the CP/UO;

Total score	*Mean*	*Variance*	*SD*
**153**	3.64	0.07	0.26

7. Procedures and recommendations for healthcare professionals / users;

Total score	*Mean*	*Variance*	*SD*
**160**	3.81	0.05	0.21

8. Training, information, and management of health workers in the pandemic era.

Total score	*Mean*	*Variance*	*SD*
**163**	3.88	0.05	0.22

Autism Spectrum Disorders CP

Total score	*Mean*	*Variance*	*SD*
634	2.81	0.02	0.14

1. Context analysis

Total score	*Mean*	*Variance*	*SD*
**69**	2.88	0.11	0.34

2. Patients access to care pathways/Operational Unit;

Total score	*Mean*	*Variance*	*SD*
**70**	3.89	0.01	0.10

3. Impact on the treatment of non-COVID patients in the CPs;

Total score	*Mean*	*Variance*	*SD*
**47**	2.61	0.23	0.48

4. Impact on the treatment of patients also SARS-COV-2 infected in the CPs;

Total score	*Mean*	*Variance*	*SD*
**23**	0.77	0.15	0.38

5. Impact of the COVID-19 pandemic on patient management;

Total score	*Mean*	*Variance*	*SD*
**98**	3.27	0.20	0.45

6. Structural and organizational changes of the CP/UO;

Total score	*Mean*	*Variance*	*SD*
**115**	3.19	0.15	0.39

7. Procedures and recommendations for healthcare professionals / users;

Total score	*Mean*	*Variance*	*SD*
**127**	3.53	0.07	0.27

8. Training, information, and management of health workers in the pandemic era.

Total score	*Mean*	*Variance*	*SD*
**85**	2.36	0.32	0.56

Diabetes CP

Total score	*Mean*	*Variance*	*SD*
660	2.90	0.06	0.25

1. Context analysis

Total score	*Mean*	*Variance*	*SD*
**54**	2.25	0.38	0.61

2. Patients access to care pathways/Operational Unit;

Total score	*Mean*	*Variance*	*SD*
**72**	4.00	0.67	0.82

3. Impact on the treatment of non-COVID patients in the CPs;

Total score	*Mean*	*Variance*	*SD*
**52**	2.89	0.48	0.69

4. Impact on the treatment of patients also SARS-COV-2 infected in the CPs;

Total score	*Mean*	*Variance*	*SD*
**20**	0.67	0.11	0.33

5. Impact of the COVID-19 pandemic on patient management;

Total score	*Mean*	*Variance*	*SD*
**87**	2.90	0.48	0.70

6. Structural and organizational changes of the CP/UO;

Total score	*Mean*	*Variance*	*SD*
**115**	3.19	0.53	0.73

7. Procedures and recommendations for healthcare professionals / users;

Total score	*Mean*	*Variance*	*SD*
**143**	3.97	0.66	0.81

8. Training, information, and management of health workers in the pandemic era.

Total score	*Mean*	*Variance*	*SD*
**119**	3.31	0.55	0.74
