# Supplementary material for: Impact of the COVID-19 Pandemic on Clinical Pathways for Non-SARS-CoV-2 Related Diseases in the Lazio Region, Italy
Source: Int J Environ Res Public Health. 2022 Jan 6;19(2):635. doi: 10.3390/ijerph19020635 (PMC8776184; doi:10.3390/ijerph19020635)
Supplement: Supplementary file 1 [file ijerph-19-00635-s001.zip › SUPPLEMENTARY TABLE S1.pdf]

Table S1. COVID SURVEY

The questionnaire consists of eight sections with 37 items. For each criterion there are 5 possible answers: yes, enough, not enough, not at all, and not applicable; each answer is connected to a percentage range (specifically: yes ≥ 75%; 51%≤ enough ≤ 74%; 26% ≤ not enough≤ 50%; and not at all ≤ 25%), and respondents are asked to indicate the verbal category that comes closest to their position.

		COVID SURVEY	yes	enough	not enough	Not at all	Not applicable
1	CONTEXT ANALYSIS	During the COVID-19 pandemic, did patients accept treatment despite the fear of contagion?					
		Compared to the same period of the previous year, during the first wave of the pandemic, did the number of accesses remain stable?					
		Compared to the first wave of the pandemic, did the number of patients undertaking the care pathways remained stable during the second wave?					
		Has the volume of procedures remained stable during first and second waves compared to the same period of the previous year?					
2	PATIENTS ACCESS TO CP/ UO	Do you use a pre-triage module during treatment?					
		Are security measures taken?					
		Are social distancing measures being taken?					
3	IMPACT ON THE TREATMENT OF NON-COVID PATIENTS IN THE [illegible]	Was the start of the treatment within the care pathway guaranteed to the patients anyway?					
		Have the cancelled visits been rescheduled and recovered?					
		Has remote monitoring been activated for patients who could not interrupt the treatment (telemedicine)?					
4	IMPACT ON THE TREATMENT OF PATIENTS ALSO SARS COV-2 INFECTED IN THE [illegible]	Have you treated patients affected by COVID-19 within the care pathway/hospital ward?					
		If yes or enough: in hospital in COVID wards?					
		If yes or enough: was it in COVID wards with telephone counselling?					
		Have the care pathways for COVID and NON_COVID patients been separated?					
5	IMPACT OF THE COVID-19 PANDEMIC ON PATIENT MANAGEMENT	Have ad hoc organizational solutions been implemented for patient management compared to the pre-pandemic era?					
		Has therapeutic continuity been ensured within the pathway care?					
		Have technological solutions, such as telemedicine, been adopted for patient follow-up?					
		Did all hospitalized patients repeat the screening test for SARS-Cov-2 several times during the hospitalization period?					

		Has the correct use of PPE (personal protective equipment) by healthcare professionals and patients been monitored?					
6	STRUCTURAL AND ORGANIZATIONAL CHANGES OF THE CP/UO	Has the care pathway/hospital ward remained unchanged from an organizational point of view?					
		Were outpatient and/or surgical activities guaranteed?					
		Has the timing of the transition of a patient from one care setting to another within the care pathway/hospital ward been respected?					
		Have there been multidisciplinary discussions about the patients' health conditions?					
		Have structural changes been made to encourage social distancing?					
		Have the services relating to non-deferrable diseases been guaranteed?					
7	PROCEDURES AND RECOMMENDATIONS FOR HEALTHCARE PROFESSIONALS / USERS	Have recommendations for the patients been made clear and visible?					
		If yes, or enough, have they been respected?					
		Have recommendations for relatives been made clear and visible?					
		If yes, or enough, have they been respected?					
		Have recommendations for healthcare professionals been made clear and visible?					
		If yes, or enough, have they been respected?					
8	TRAINING, INFORMATION AND MANAGEMENT OF HEALTH WORKERS IN THE PANDEMIC ERA	Have health care workers involved in care pathways/hospital wards been trained on the dressing-doffing PPE procedures?					
		Has the exposed health care personnel been periodically subjected to rhino-pharyngeal swabs to evaluate the possible positivity for SARS-CoV-2?					
		Has the staff been equipped with PPE of modulated efficiency with respect to the professional risk to which they have been exposed?					
		Have dirty paths and clean access paths to clinical departments been organized?					
		Has the corporate anti-COVID vaccination program been performed using the employee booking portal?					
		In the company/facility, was the anti-COVID19 vaccination campaign preceded by an information campaign on the technical characteristics, methods of setting up and administering the vaccine?					
