# Supplementary material for: Impact of the COVID-19 Pandemic on Clinical Pathways for Non-SARS-CoV-2 Related Diseases in the Lazio Region, Italy
Source: Int J Environ Res Public Health. 2022 Jan 6;19(2):635. doi: 10.3390/ijerph19020635 (PMC8776184; doi:10.3390/ijerph19020635)
Supplement: Supplementary file 1 [file ijerph-19-00635-s001.zip › SUPPLEMENTARY TABLE S2.pdf]

Table S2: Clinical Pathways' data

S2a: HEART FAILURE

COVID SURVEY		CLINICAL PATHWAY – HEART FAILURE
CONTEXT ANALYSIS	During the COVID-19 pandemic, did patients accept treatment despite the fear of contagion?	20,0% / 60,0% / 20,0%
	Compared to the same period of the previous year, during the first wave of the pandemic, did the number of accesses remain stable?	60,0% / 20,0% / 20,0%
	Compared to the the first wave of the pandemic, did the number of patients undertaking the care pathways remained stable during the second wave?	80,0% / 20,0%
	Has the volume of procedures remained stable during first and second waves compared to the same period of the previous year?	20,0% / 60,0% / 20,0%
PATIENTS ACCESS TO CP/UO	Do you use a pre-triage module during treatment?	60,0% / 40,0%
	Are security measures taken?	60,0% / 40,0%
	Are social distancing measures taken?	60,0% / 20,0% / 20,0%
IMPACT ON THE TREATMENT OF NON-COVID PATIENTS IN THE CLINICAL PATHWAY	Was the start of the treatment within the care pathway garanteed to the patients anyway?	80,0% / 20,0%
	Have the cancelled visits been rescheduled and recovered?	20,0% / 20,0% / 20,0% / 40,0%
	Has remote monitoring been activated for patients who could not interrupt the treatment path (telemedicine)?	20,0% / 20,0% / 20,0% / 40,0%
IMPACT ON THE TREATMENT OF PATIENTS ALSO SARS COV-2 INFECTED IN THE CLINCAL PATHWAY	Have you treated patients affected by COVID-19 within the care pathway/hospital ward?	40,0% / 40,0% / 20,0%
	If yes or enough: in hospital in COVID wards?	100,0%
	If yes or enough: was it in COVID wards with telephone counselling?	100,0%
	Have the clinical-assistance paths for COVID and NON_COVID patients been separated?	80,0% / 20,0%
IMPACT OF THE COVID-19 PANDEMIC ON PATIENT MANAGEMENT	Have ad hoc organizational solutions been implemented for patient management with respect to the pre-pandemic era?	60,0% / 20,0% / 20,0%
	Has therapeutic continuity been ensured within the pathway care?	80,0% / 20,0%
	Have technological solutions, such as telemedicine, been adopted for patient follow-up?	20,0% / 40,0% / 40,0%
	Did all hospitalized patients repeat the screening test for SARS-Cov-2 several times during the hospitalization period?	60,0% / 20,0% / 20,0%
	Has the correct use of PPE (personal protective equipment) by healthcare professionals and patients been monitored?	80,0% / 20,0%

Section	Question	Yes	enough	not enough	Not at all	Not applicable
STRUCTURAL AND ORGANIZATIONAL CHANGES OF THE CP/UO	Has the care pathway/hospital ward remained unchanged from an organizational point of view?		40,0%	20,0%	20,0%	20,0%
	Have outpatient and/or surgical activities been guaranteed?	60,0%			20,0%	20,0%
	Has the timing of the transition of a patient from one care setting to another within the care pathway/hospital ward been respected?	20,0%	20,0%		20,0%	40,0%
	Have there been multidisciplinary discussions about the patients' health conditions?		40,0%	20,0%		40,0%
	Have structural changes been made to encourage social distancing?	60,0%	20,0%		20,0%	
	Have the services relating to non-deferrable diseases been guaranteed?	80,0%			20,0%	
PROCEDURES AND RECOMMENDATIONS FOR HEALTHCARE PROFESSIONALS / USERS	Have recommendations for the patients been made clear and visible?	40,0%	40,0%		20,0%	
	If yes, or enough, have they been respected?	20,0%	80,0%			
	Have recommendations for relatives been made clear and visible?	60,0%	20,0%		20,0%	
	If yes, or enough, have they been respected?	40,0%	60,0%			
	Have the recommendations been made clear and visible to healthcare professionals?	60,0%	20,0%		20,0%	
	If yes, or enough, have they been respected?	60,0%	20,0%		20,0%	
TRAINING, INFORMATION AND MANAGEMENT OF HEALTH WORKERS IN THE PANDEMIC ERA	Have health care workers involved in care pathways/hospital wards been trained on the dressing-doffing PPE procedures?	80,0%	20,0%			
	Has the exposed health care personnel been periodically subjected to rhino- pharyngeal swabs to evaluate the possible positivity for SARS-CoV-2?	60,0%	40,0%			
	Has the staff been equipped with PPE of modulated efficiency with respect to the professional risk to which they have been exposed?	80,0%	20,0%			
	Have dirty paths and clean access paths to clinical departments been organized?	40,0%	60,0%			
	Has the corporate anti-COVID vaccination program been performed using the employee booking portal?	40,0%			40,0%	20,0%
	In the company/facility, was the anti-COVID19 vaccination campaign preceded by an information campaign on the technical characteristics, methods of setting up and administering the vaccine?	60,0%			20,0%	20,0%

Legend: Yes | enough | not enough | Not at all | Not applicable

S2b: HEREDITARY BREAST AND OVARIAN CANCER

COVID SURVEY		CLINICAL PATHWAY<br>HEREDITARY BREAST AND OVARIAN CANCER
CONTEXT ANALYSIS	During the COVID-19 pandemic, did patients accept treatment despite the fear of contagion?	28,6% / 42,9% / 14,3% / 14,3%
	Compared to the same period of the previous year, during the first wave of the pandemic, did the number of accesses remain stable?	14,3% / 42,9% / 28,6% / 14,3%
	Compared to the the first wave of the pandemic, did the number of patients undertaking the care pathways remained stable during the second wave?	85,7% / 14,3%
	Has the volume of procedures remained stable during first and second waves compared to the same period of the previous year?	42,9% / 28,6% / 28,6%
PATIENTS ACCESS TO CP/UO	Do you use a pre-triage module during treatment?	71,4% / 14,3% / 14,3%
	Are security measures taken?	100,0%
	Are social distancing measures taken?	85,7% / 14,3%
IMPACT ON THE TREATMENT OF NON-COVID PATIENTS IN THE CLINICAL PATHWAY	Was the start of the treatment within the care pathway garanteed to the patients anyway?	85,7% / 14,3%
	Have the cancelled visits been rescheduled and recovered?	85,7% / 14,3%
	Has remote monitoring been activated for patients who could not interrupt the treatment path (telemedicine)?	28,6% / 28,6% / 14,3% / 28,6%
IMPACT ON THE TREATMENT OF PATIENTS ALSO SARS COV-2 INFECTED IN THE CLINCAL PATHWAY	Have you treated patients affected by COVID-19 within the care pathway/hospital ward?	14,3% / 42,9% / 42,9%
	If yes or enough: in hospital in COVID wards?	14,3% / 85,7%
	If yes or enough: was it in COVID wards with telephone counselling?	14,3% / 85,7%
	Have the clinical-assistance paths for COVID and NON_COVID patients been separated?	57,1% / 14,3% / 28,6%
IMPACT OF THE COVID-19 PANDEMIC ON PATIENT MANAGEMENT	Have ad hoc organizational solutions been implemented for patient management with respect to the pre-pandemic era?	100,0%
	Has therapeutic continuity been ensured within the pathway care?	100,0%
	Have technological solutions, such as telemedicine, been adopted for patient follow-up?	14,3% / 14,3% / 42,9% / 28,6%
	Did all hospitalized patients repeat the screening test for SARS-Cov-2 several times during the hospitalization period?	85,7% / 14,3%
	Has the correct use of PPE (personal protective equipment) by healthcare professionals and patients been monitored?	85,7% / 14,3%

Area	Question	Yes	enough	not enough	Not at all	Not applicable
STRUCTURAL AND ORGANIZATIONAL CHANGES OF THE CP/UO	Has the care pathway/hospital ward remained unchanged from an organizational point of view?	57,1%	28,6%	14,3%		
	Have outpatient and/or surgical activities been guaranteed?	71,4%	28,6%			
	Has the timing of the transition of a patient from one care setting to another within the care pathway/hospital ward been respected?	71,4%	28,6%			
	Have there been multidisciplinary discussions about the patients' health conditions?	85,7%	14,3%			
	Have structural changes been made to encourage social distancing?	71,4%	14,3%			14,3%
	Have the services relating to non-deferrable diseases been guaranteed?	85,7%	14,3%			
PROCEDURES AND RECOMMENDATIONS FOR HEALTHCARE PROFESSIONALS / USERS	Have recommendations for the patients been made clear and visible?	100,0%				
	If yes, or enough, have they been respected?	71,4%	28,6%			
	Have recommendations for relatives been made clear and visible?	100,0%				
	If yes, or enough, have they been respected?	71,4%	28,6%			
	Have the recommendations been made clear and visible to healthcare professionals?	100,0%				
	If yes, or enough, have they been respected?	85,7%				14,3%
TRAINING, INFORMATION AND MANAGEMENT OF HEALTH WORKERS IN THE PANDEMIC ERA	Have health care workers involved in care pathways/hospital wards been trained on the dressing-doffing PPE procedures?	100,0%				
	Has the exposed health care personnel been periodically subjected to rhino- pharyngeal swabs to evaluate the possible positivity for SARS-CoV-2?	100,0%				
	Has the staff been equipped with PPE of modulated efficiency with respect to the professional risk to which they have been exposed?	85,7%	14,3%			
	Have dirty paths and clean access paths to clinical departments been organized?	85,7%				14,3%
	Has the corporate anti-COVID vaccination program been performed using the employee booking portal?	100,0%				
	In the company/facility, was the anti-COVID19 vaccination campaign preceded by an information campaign on the technical characteristics, methods of setting up and administering the vaccine?	100,0%				

S2c: AUTISM SPECTRUM DISORDER

COVID SURVEY		CLINICAL PATHWAY AUTISM SPECTRUM DISORDER			
CONTEXT ANALYSIS	During the COVID-19 pandemic, did patients accept treatment despite the fear of contagion?	66,7%	33,3%		
	Compared to the same period of the previous year, during the first wave of the pandemic, did the number of accesses remain stable?	16,7%	66,7%	16,7%	
	Compared to the the first wave of the pandemic, did the number of patients undertaking the care pathways remained stable during the second wave?	50,0%	16,7%	16,7%	16,7%
	Has the volume of procedures remained stable during first and second waves compared to the same period of the previous year?	66,7%	16,7%	16,7%	
PATIENTS ACCESS TO CP/ UO	Do you use a pre-triage module during treatment?	100,0%			
	Are security measures taken?	100,0%			
	Are social distancing measures taken?	66,7%	33,3%		
IMPACT ON THE TREATMENT OF NON-COVID PATIENTS IN THE CLINICAL PATHWAY	Was the start of the treatment within the care pathway garanteed to the patients anyway?	83,3%	16,7%		
	Have the cancelled visits been rescheduled and recovered?	33,3%	33,3%	33,3%	
	Has remote monitoring been activated for patients who could not interrupt the treatment path (telemedicine)?	16,7%	33,3%	50,0%	
IMPACT ON THE TREATMENT OF PATIENTS ALSO SARS COV-2 INFECTED IN THE CLINCAL PATHWAY	Have you treated patients affected by COVID-19 within the care pathway/hospital ward?	100,0%			
	If yes or enough: in hospital in COVID wards?	33,3%	66,7%		
	If yes or enough: was it in COVID wards with telephone counselling?	33,3%	66,7%		
	Have the clinical-assistance paths for COVID and NON_COVID patients been separated?	50,0%	16,7%	33,3%	
IMPACT OF THE COVID-19 PANDEMIC ON PATIENT MANAGEMENT	Have ad hoc organizational solutions been implemented for patient management with respect to the pre-pandemic era?	83,3%	16,7%		
	Has therapeutic continuity been ensured within the pathway care?	83,3%	16,7%		
	Have technological solutions, such as telemedicine, been adopted for patient follow-up?	50,0%	33,3%	16,7%	
	Did all hospitalized patients repeat the screening test for SARS-Cov-2 several times during the hospitalization period?	33,3%	16,7%	50,0%	
	Has the correct use of PPE (personal protective equipment) by healthcare professionals and patients been monitored?	100,0%			

STRUCTURAL AND ORGANIZATIONAL CHANGES OF THE CP/UO	Has the care pathway/hospital ward remained unchanged from an organizational point of view?	16,7% · 66,7% · 16,7%
	Have outpatient and/or surgical activities been guaranteed?	50,0% · 33,3% · 16,7%
	Has the timing of the transition of a patient from one care setting to another within the care pathway/hospital ward been respected?	33,3% · 50,0% · 16,7%
	Have there been multidisciplinary discussions about the patients' health conditions?	83,3% · 16,7%
	Have structural changes been made to encourage social distancing?	50,0% · 33,3% · 16,7%
	Have the services relating to non-deferrable diseases been guaranteed?	100,0%
PROCEDURES AND RECOMMENDATIONS FOR HEALTHCARE PROFESSIONALS / USERS	Have recommendations for the patients been made clear and visible?	100,0%
	If yes, or enough, have they been respected?	33,3% · 66,7%
	Have recommendations for relatives been made clear and visible?	100,0%
	If yes, or enough, have they been respected?	33,3% · 50,0% · 16,7%
	Have the recommendations been made clear and visible to healthcare professionals?	83,3% · 16,7%
	If yes, or enough, have they been respected?	83,3% · 16,7%
TRAINING, INFORMATION AND MANAGEMENT OF HEALTH WORKERS IN THE PANDEMIC ERA	Have health care workers involved in care pathways/hospital wards been trained on the dressing-doffing PPE procedures?	66,7% · 33,3%
	Has the exposed health care personnel been periodically subjected to rhino- pharyngeal swabs to evaluate the possible positivity for SARS-CoV-2?	83,3% · 16,7%
	Has the staff been equipped with PPE of modulated efficiency with respect to the professional risk to which they have been exposed?	66,7% · 16,7% · 16,7%
	Have dirty paths and clean access paths to clinical departments been organized?	33,33% · 66,67%
	Has the corporate anti-COVID vaccination program been performed using the employee booking portal?	16,7% · 16,7% · 66,7%
	In the company/facility, was the anti-COVID19 vaccination campaign preceded by an information campaign on the technical characteristics, methods of setting up and administering the vaccine?	66,7% · 16,7% · 16,7%

Yes · enough · not enough · Not at all · Not applicable

S2d: DIABETES

COVID SURVEY		CLINICAL PATHWAY DIABETES
CONTEXT ANALYSIS	During the COVID-19 pandemic, did patients accept treatment despite the fear of contagion?	16,7% · 66,7% · 16,7%
	Compared to the same period of the previous year, during the first wave of the pandemic, did the number of accesses remain stable?	100,0%
	Compared to the the first wave of the pandemic, did the number of patients undertaking the care pathways remained stable during the second wave?	100,0%
	Has the volume of procedures remained stable during first and second waves compared to the same period of the previous year?	100,0%
PATIENTS ACCESS TO CP/UO	Do you use a pre-triage module during treatment?	100,0%
	Are security measures taken?	100,0%
	Are social distancing measures taken?	100,0%
IMPACT ON THE TREATMENT OF NON-COVID PATIENTS IN THE CLINICAL	Was the start of the treatment within the care pathway garanteed to the patients anyway?	16,7% · 50,0% · 16,7% · 16,7%
	Have the cancelled visits been rescheduled and recovered?	83,3% · 16,7%
	Has remote monitoring been activated for patients who could not interrupt the treatment path (telemedicine)?	83,3% · 16,7%
IMPACT ON THE TREATMENT OF PATIENTS ALSO SARS COV-2 INFECTED IN THE CLINCAL PATHWAY	Have you treated patients affected by COVID-19 within the care pathway/hospital ward?	16,7% · 16,7% · 33,3% · 33,3%
	If yes or enough: in hospital in COVID wards?	16,7% · 83,3%
	If yes or enough: was it in COVID wards with telephone counselling?	16,7% · 16,7% · 66,7%
	Have the clinical-assistance paths for COVID and NON_COVID patients been separated?	16,7% · 16,7% · 66,7%
IMPACT OF THE COVID-19 PANDEMIC ON PATIENT MANAGEMENT	Have ad hoc organizational solutions been implemented for patient management with respect to the pre-pandemic era?	100,0%
	Has therapeutic continuity been ensured within the pathway care?	66,7% · 33,3%
	Have technological solutions, such as telemedicine, been adopted for patient follow-up?	83,3% · 16,7%
	Did all hospitalized patients repeat the screening test for SARS-Cov-2 several times during the hospitalization period?	100,0%
	Has the correct use of PPE (personal protective equipment) by healthcare professionals and patients been monitored?	100,0%

Section	Question	Yes	enough	not enough	Not at all	Not applicable
STRUCTURAL AND ORGANIZATIONAL CHANGES OF THE CP/UO	Has the care pathway/hospital ward remained unchanged from an organizational point of view?	83,3%	16,7%			
	Have outpatient and/or surgical activities been guaranteed?	50,0%	33,3%			16,7%
	Has the timing of the transition of a patient from one care setting to another within the care pathway/hospital ward been respected?	16,7%	66,7%			16,7%
	Have there been multidisciplinary discussions about the patients' health conditions?	16,7%	50,0%	16,7%		16,7%
	Have structural changes been made to encourage social distancing?	100,0%				
	Have the services relating to non-deferrable diseases been guaranteed?	66,7%	16,7%			16,7%
PROCEDURES AND RECOMMENDATIONS FOR HEALTHCARE PROFESSIONALS / USERS	Have recommendations for the patients been made clear and visible?	100,0%				
	If yes, or enough, have they been respected?	83,3%	16,7%			
	Have recommendations for relatives been made clear and visible?	100,0%				
	If yes, or enough, have they been respected?	100,0%				
	Have the recommendations been made clear and visible to healthcare professionals?	100,0%				
	If yes, or enough, have they been respected?	100,0%				
TRAINING, INFORMATION AND MANAGEMENT OF HEALTH WORKERS IN THE PANDEMIC ERA	Have health care workers involved in care pathways/hospital wards been trained on the dressing-doffing PPE procedures?	100,0%				
	Has the exposed health care personnel been periodically subjected to rhino- pharyngeal swabs to evaluate the possible positivity for SARS-CoV-2?	100,0%				
	Has the staff been equipped with PPE of modulated efficiency with respect to the professional risk to which they have been exposed?	83,3%	16,7%			
	Have dirty paths and clean access paths to clinical departments been organized?					100,0%
	Has the corporate anti-COVID vaccination program been performed using the employee booking portal?	100,0%				
	In the company/facility, was the anti-COVID19 vaccination campaign preceded by an information campaign on the technical characteristics, methods of setting up and administering the vaccine?	100,0%				

Yes enough not enough Not at all Not applicable
